# Pleuro-pulmonary ultrasound in the diagnosis and follow-up of lung infections in children with cancer: a pilot study

**DOI:** 10.1007/s40477-021-00650-3

**Published:** 2022-03-09

**Authors:** Mariaclaudia Meli, Milena La Spina, Luca Lo Nigro, Gian Luca Trobia, Giovanna Russo, Andrea Di Cataldo

**Affiliations:** 1 Pediatric Hematology and Oncology Unit, Department of Clinical and Experimental Medicine, School of Medicine, University of Catania, Via Santa Sofia 78, 95123 Catania, Italy; 2 Pediatric and Pediatric Emergency Room, Cannizzaro Emergency Hospital, Catania, Italy

**Keywords:** Childhood cancer, Lung infection, Pneumonia, Febrile neutropenia, Lung ultrasound

## Abstract

**Purpose:**

Febrile neutropenia and lung infections are common and potential fatal complications of pediatric cancer patients during chemotherapy. Lung ultrasound (LUS) has a good accuracy in the diagnosis of pneumonia in childhood, but there is no data concerning its use in the diagnosis and follow-up of pulmonary infection in children with cancer. The goal of this pilot study is to verify the feasibility of lung ultrasonography for the diagnosis and follow up of pneumonia in children and adolescents with cancer.

**Material and methods:**

This is a prospective observational case–control monocentric study conducted in the Pediatric Hematology and Oncology Department of University Hospital of Catania in patients aged < 18 years with cancer. Attending Physician used ultrasonography to detect pneumonia in cancer children with fever. As control group, cancer patients with no infection suspicion were also tested. LUS results were compared to chest X-ray (CXR) and/or chest CT scan, when these imaging techniques were performed, according to clinical indication.

**Results:**

Thirty-eight patients were studied. All underwent LUS, 16 underwent CXR, 3 chest CT. Statistical analysis showed LUS specificity of 93% (95% CI 84–100%), and sensitivity of 100%; CXR, instead, showed a specificity of 83% (95% CI 62–100%) and a sensitivity of 50% (95% CI 1–99%).

**Conclusion:**

This study shows for the first time that LUS allows physicians to diagnose pneumonia in children and young adults with cancer, with high specificity and sensitivity.

**Supplementary Information:**

The online version contains supplementary material available at 10.1007/s40477-021-00650-3.

## Introduction

The incidence of pneumonia in cancer patients varies from 17 to 24% and clinical response to specific treatment varies from 60 to 65% with an infection-related mortality of 38% [1–3]. Pneumonia accounts for as much as 50% of septic shock cases in cancer patients.

Febrile neutropenia is one of the most frequent complications in cancer patients and sometimes, if not promptly treated, has unfavourable prognosis with possible evolution towards septic shock, acute organ dysfunction, disseminated intravascular coagulation and eventually death. The lungs are one of the most frequent sites of infection in oncological patients during neutropenia [1–4].

Diagnostic exams such as CXR and CT are often required in febrile cancer patients. However, CXR, especially during neutropenia, is not very specific. It is unable to make differential diagnosis between bacterial, viral or mycotic pneumonia, and for this reason, in the suspicion of a fungal etiology (*Candida* or *Aspergillus*) patients undergo chest CT scan, that has higher specificity. Moreover, CXR has low sensitivity in the initial phase of infection, particularly if performed in single projection [5, 6]. The literature has also already established the inconsistent role of CXR in the diagnosis of lung infection in the neutropenic patient probably due to the low number of neutrophils involved in the production of the inflammatory response [4]. On the other hand, chest CT scan is complicated by organizational difficulties and greater exposure to ionizing radiation, especially in cancer patients already exposed to multiple radiological examinations [7]. For all these reasons, we need new methods for a correct and quick diagnosis of pneumonia.

Lung ultrasound (LUS) has been proposed as an alternative first-line imaging modality to diagnose community acquired pneumonia in children, with promising results [8]. There is evidence that LUS may have greater sensitivity, similar specificity and better inter-operator reliability in the diagnosis of pneumonia when compared with CXR [9–11]. Moreover, LUS is radiation free and can be performed at bedside with better manouvrability and efficacy than CXR. Moreover, LUS has also been shown to be more sensitive than CXR in identifying sub-centimeter consolidations with air bronchograms often not evident on CXR [12].

**Fig. 1:**
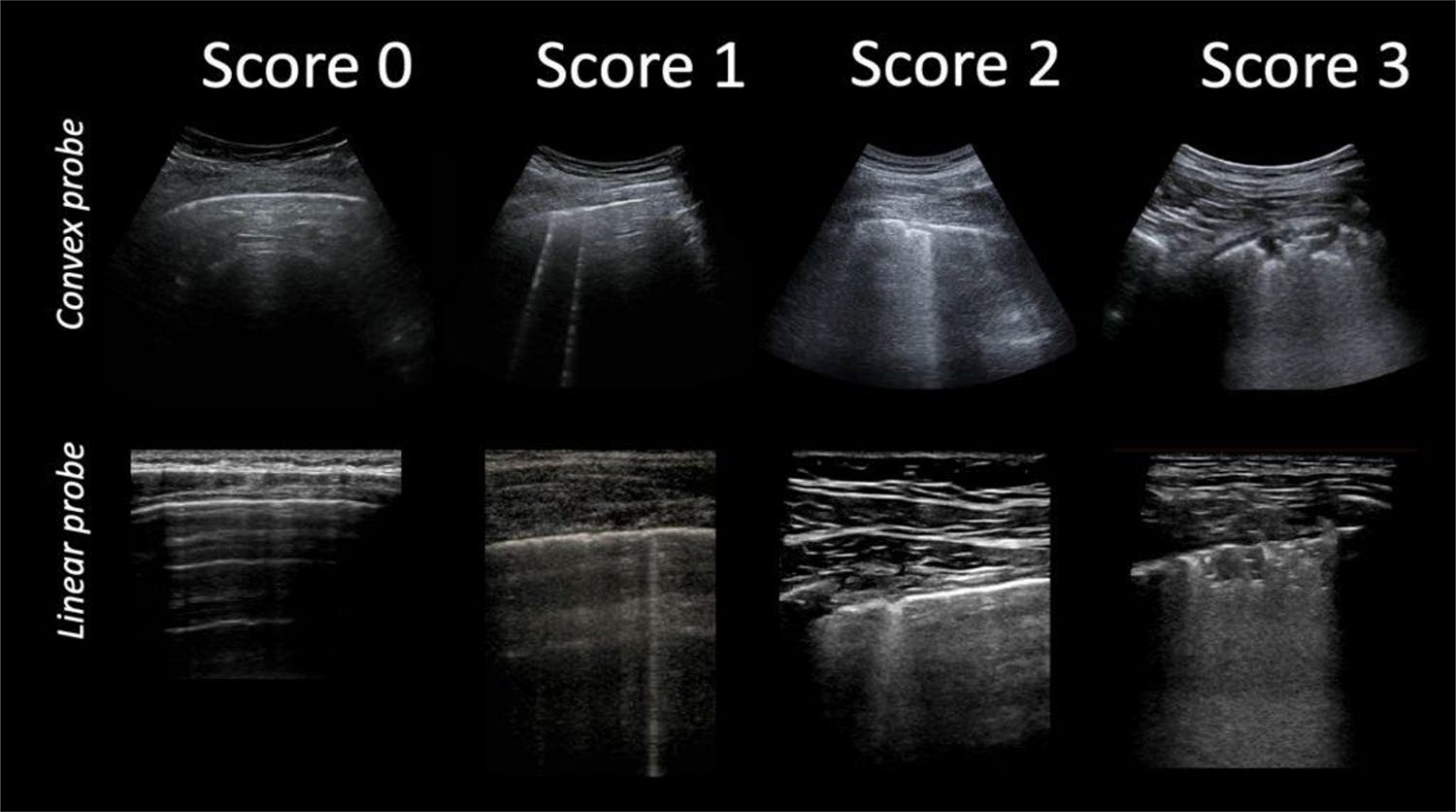
LUS score

Consolidations appear on LUS as hypoechoic subpleural areas, with interruption of the overlying pleural line [13, 14]. The presence of compact vertical artifacts is frequent expression of wall reinforcements typically produced by areas with fluid content [15, 16]. Interstitial pneumonia is depicted by B lines (vertical lines), either isolated or confluent to appear like a white lung pattern based on gravity (Fig. 1).

Despite the large literature in the pediatric field on the role of LUS in the diagnosis of pneumonia, to our knowledge there are no studies concerning its utilization in the diagnosis and follow-up of pulmonary infection in children with cancer.

In our Unit of Pediatric Hematology and Oncology we recently introduced the routine use of bedside LUS, as an aid to physical examination, in order to evaluate pulmonary involvement and subsequent clinical management in both in- and out-patients with fever. Recently during the COVID-19 pandemic, this technique turned out to be particularly useful in the diagnostic management of our patients, to optimize diagnostic and therapeutic choices.

The goal of this study is to explore the use of LUS as a tool for the diagnosis of pneumonia in children with cancer, neutropenic and non neutropenic.

The primary objective of our study is to evaluate the diagnostic accuracy of LUS in pediatric cancer patients, especially during neutropenia. We want to evaluate whether ultrasound sensitivity is unaffected by the low neutrophil count, similarly to what happens with chest CT scan, or, alternatively wheteher LUS sensititivity is lowered by concurrent neutropenia, similarly to CXR. Furthermore, we want to evaluate whether the underlying disease, the therapies administered (chemotherapy and radiotherapy) and the state of neutropenia can be factors influencing the sensitivity and specificity of the ultrasound examination.

The secondary objectives of the study are to evaluate the role of LUS in the follow-up of patients with lung infection and the tolerability of LUS, taking into account child's age and his psycho-physical health.

## Methods and analysis

This prospective observational study was performed at the Unit of Pediatric Hematology and Oncology in Catania between March and June 2020, at the beginning of the spread of the COVID-19 epidemic in Italy. It was used as a complementary technique to the physical examination, with the aim of limiting daily out-patient access or in-ward admission and transfer of patients to other departments to perform tests. All admitted cancer patients undergoing therapy (steroid, chemotherapy, radiotherapy) with suspected infection in progress, participated in the study. As a case–control, a similar number of patients, with no signs of infection, randomly picked, underwent LUS. The protocol was approved by the internal Institutional Review Board (ethical approval code: 01/2020), after consideration by the heads of Pediatric Hemato-Oncology and Radiology Units, plus all physicians and nurses of the same units. The performed procedures were in accordance with the principles of the 1964 Declaration of Helsinki and its later amendments (2013). Informed consent was obtained from all participants.

The inclusion criteria were age 0–18 years, diagnosis of leukemia or solid tumor, therapy in progress. Patients with the following features were excluded: ongoing asthma crisis, cystic fibrosis, bronchodysplasia, congenital cardio-pulmonary malformation, primary and metastatic pleuro-pulmonary tumor localization.

Infection was defined as: body temperature (T) greater than or equal to 38 °C and increased c-reactive protein (CRP) (normal range 0–5 mg/dl) and/or procalcitonin (normal range 0–0 ng/ml), with or without respiratory signs and symptoms [cough, tachydispnea, SaO_2_ < 96%, rales, reduction of vesicular murmur (VM)].

For each patient we assessed age, sex, underlying cancer, the absolute number of white blood cells and neutrophils at the time of the LUS, distinguishing the patients in:

- Neutropenic (neutrophils less than or equal to 1000/mmc).
- Non-neutropenic (neutrophils greater than 1000/mmc).

We reported the presence or absence of fever (*T* ≥ 38 °C), respiratory symptoms and signs, the results of hematological tests for infection and COVID-19 nose-pharingeal swabs result. CXR and/or chest CT scans were also recorded, if perforemd.

CXR and chest CT were evaluated with radiologists and considered positive in the presence of pulmonary thickening or marked accentuation of the bronchovascular texture.

These tests were performed only if considered useful and appropriate for diagnostic purposes and clinical management.

LUS was always performed by two operators: a pediatrician with a 6-month ultrasound training, and an expert sonographer pediatrician who reviewed all exams with a 5–10 MHz linear probe or with a convex probe in obese patients or teenagers. The probe was placed perpendicularly, oblique and parallel to the ribs in the anterior, lateral and posterior thorax as described by Copetti and Cattarossi [17] with the patient supine and seated to scan the posterior thorax. The sonographer was unaware of the CRX results.

Pneumonia was diagnosed in the presence of lung consolidation, air or fluid bronchograms in the sub-pleural region > 1 cm, multiple air or fluid bronchograms, air bronchogram < 1 cm with multiple B lines in the neighboring sites, confluent B lines or white lung as previously classified [2, 18, 19].

All ultrasound examinations including A-lines only, rare B-lines (less than 3 per ultrasound scan) or single and isolated consolidation < 1 cm were considered normal.

In cases of positive LUS, follow-up was performed to evaluate the evolution of the described picture after 3 and 7 days. In case of persistent positive LUS, a monthly sonography was performed.

The compliance of children during the ultrasound examination by assigning a score from 0 to 2 was also evaluated:

- 0 If he was uncooperative (if the patient cried or refused to undergo the exam),
- 1 If he was indifferent during the exam,
- 2 If he was proactive (took the exam as a game, participated curiously in the exam).

We divided the recruited patients into four groups:

1. Non-infected non-neutropenic patients: absolute number of neutrophils > 1000/mmc, no fever, normal CRP and/or procalcitonin,
2. Non-infected neutropenic patients: absolute number of neutrophils ≤ 1000/mmc, no fever, normal CRP and/or procalcitonin,
3. Infected non-neutropenic patients: absolute number of neutrophils > 1000/mmc, fever, positive CRP and/or procalcitonin,
4. Infected neutropenic patients: absolute number of neutrophils ≤ 1000/mmc, fever, positive CRP and/or procalcitonin.

Patients of group 1 and 2 did not show signs of ongoing infection and LUS results were analyzed in order to identify if there was an increase in false positives related to the underlying disease, the treatments administered for cancer or the number of white blood cells and neutrophils in patients without infection signs.

LUS results of patients who underwent also CXR and/or CT scan were analyzed in order to evaluate the sensitivity of LUS compared to CXR and CT images.

We considered the presence of fever associated with increased inflammation indices (pcr and/or pct) and positive CXR as the standard for calculating specificity and sensitivity; we calculated also the 95% confidence intervals (CI) when applicable.

## Results

We recruited 38 patients, 23 (60%) male and 15 (40%) female. Diagnosis was acute leukemia (AL) in 22, brain tumor in 5, lymphoma in 4, sarcoma in 3 [1 renal sarcoma (RS), 1 rhabdomyosarcoma (RMS), 1 osteosarcoma (OS)], Wilms tumor (WT) in 2, neuroblastoma (NBL) in 1 and desmoid fibromatosis in 1 patient. The mean age was 9.2 years and the median 9 years.

All febrile and neutropenic patients underwent blood cultural tests (Table 2) and all patients underwent COVID-19 nose-pharyngeal swabs with negative result.

All patients underwent LUS, 16 underwent CXR, 3 chest CT.

Group 1 included 8 cases (21%) of non-infected non-neutropenic patients, mean age 10.3 years, median 9.5 years. LUS was negative in all 8 patients. CXR was performed in four cases, as part of the initial evaluation and stadiation at onset of cancer, and was negative in all cases (Table 1).

**Table 1:** Group 1, 2 and 3 features

Patient	Sex	Age	Type of cancer	Symptoms	Leucocytes	Neutrophils	PCR/PCT	LUS	CXR	CT
Group 1										
1	M	14	AML	–	227.000	7.720	Neg	NEG	NEG	–
2	M	16	ALL	–	279.000	5.820	Neg	NEG	NEG	–
3	M	10	ALL	–	2.800	1.624	Neg	NEG	NEG	–
4	F	16	ALL	–	8.860	8.150	Neg	NEG	–	–
5	F	9	MB	–	2.370	1.710	Neg	NEG	–	–
6	M	7	ALL	–	3.620	2.330	Neg	NEG	–	–
7	M	4	NBL	–	2.760	1.640	Neg	NEG	–	–
8	M	7	ALL	–	325.120	130.000	Neg	NEG	NEG	–
Group 2										
9	M	5	ALL	–	1.660	400	Neg	NEG	POS	–
10	M	10	AML	–	100	0	Neg	NEG	–	–
11	M	2	ALL	–	1.280	10	Neg	NEG		–
12	M	5	ALL	–	4.030	200	Neg	NEG	–	–
13	F	8	DESMOID	–			Neg	NEG	–	–
14	M	11	ALL	–	10.180	380	Neg	NEG	NEG	–
15	M	3	ALL	–	2.830	970	Neg	NEG	NEG	–
16	F	4	ALL	–	6.750	140	Neg	POS	POS	–
17	M	3	WT	–	650	470	Neg	NEG	–	–
18	F	7	GLIOMA	–	3.960	1.170	Neg	NEG	–	–
19	M	17	AML	–	1.660	1.000	Neg	NEG	NEG	–
20	F	2	ALL	–	3.260	610	Neg	NEG	NEG	–
21	F	16	ALL		550	5	Neg	NEG	–	–
Group 3										
22	F	9	NHL	Absent	3.810	3.170	105	NEG	NEG	–
23	M	16	GLIOMA	Absent	18.040	14.460	110/neg	NEG	NEG	–
24	M	16	GLIOMA	Cought riduction of VM	14.180	11.200	105/3.5	POS	–	–
25	M	16	GLIOMA	Late onset cough, SO_2_ < 96%, wheezing, reduction oF VM	8.030	4.860	183/1.3	POS	NEG Second time: POS	–
26	M	8	AML	Late onset Cough, reduction of VM	6.760	3.180	28/neg	POS	NEG	POS
27	M	9	OS	Cough, reduction of VM	4.660	3.542	105/1.1	POS	POS	POS
28	F	3	ALL	Cough, SO_2_ < 96%, wheezing, reduction of VM	5.600	2.800	242/1.1	POS	POS	POS

Group 2 included 13 (34%) non-infected neutropenic patients, mean age 7.1 years, median 5 years. LUS was negative in 12 patients; in one case, it revealed rare B lines and bilateral subpleural air bronchograms;

CXR was performed in 6 cases, with negative results in four cases and positive findings in the remaing 2: particularly, in 1 case, CXR showed signs of leukemia infiltration, at onset of disease, in agreement with LUS findings; in the other one, radiogram revealed nuanced hilar thickening, not evidenced on LUS.

The concordance between ultrasound and CXR was 83% (Table 1).

**Fig. 2:**
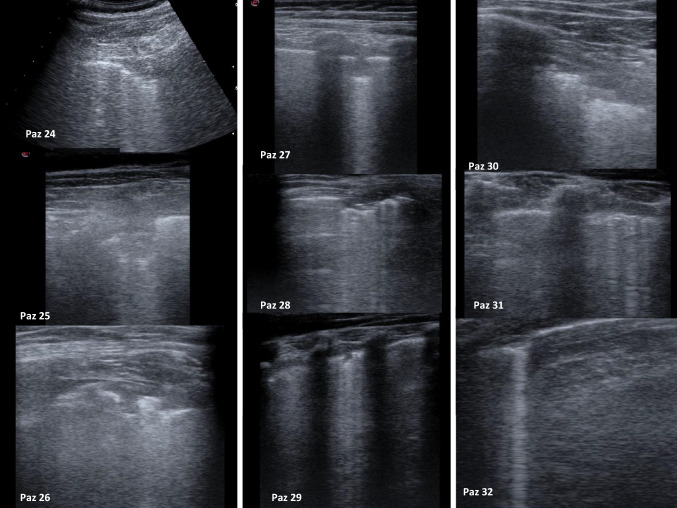
Pathological images at LUS

Group 3 included 7 infected non-neutropenic patients (19%), mean age 11 years, median 9 years. Three patients (42%) presented with respiratory symptoms; two initially asymptomatic patients subsequently presented respiratory symptoms within 24 h from initial evaluation (cases 25 and 26) (Table 1). In all these five patients LUS revealed abnormal findings (Fig. 2).

Six CXR were performed: 4 were negative and 2 were positive. Particularly, it was negative in cases 25 and 26 at onset of fever; nontheless, a second CXR was repeated in case 25 (Table1) 4 days after the onset of respiratory symptoms and turned out positive.

**Fig. 3:**
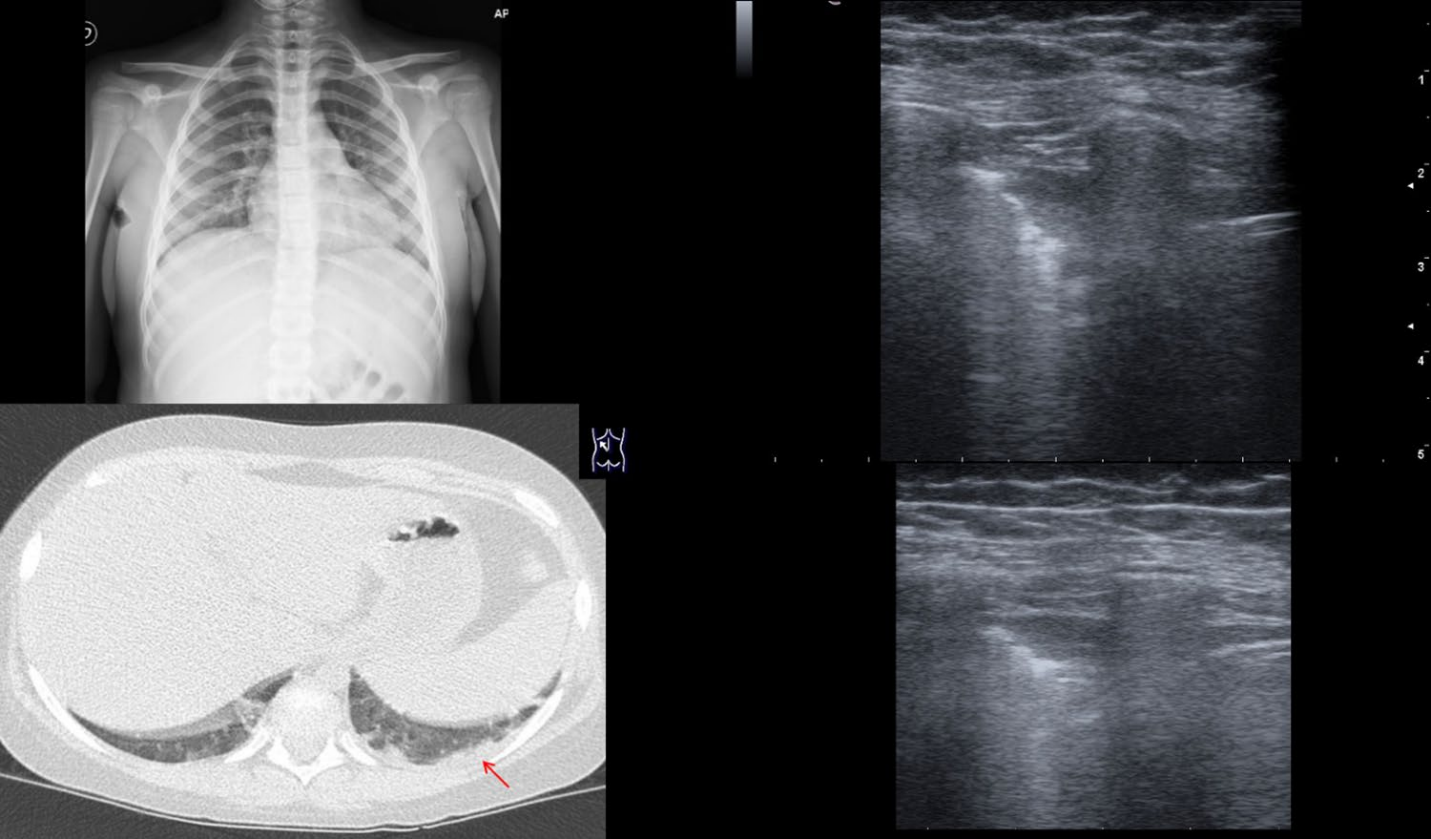
CXR, TC and lus of case 26

Chest CT scan was performed in 3 patients, with positive results in all of them. Particularly, also case 26, with positive LUS and negative CXR, revealed pneumonia findings (Fig. 3, ESM Video 1).

There was concordance between LUS and CXR in 67% of cases, between LUS and CT scan in 100%.

In one of the three cases (33%) chest CT was discordant with CXR.

Group 4 included ten infected neutropenic patients (26%), mean age 8.5 years, median 10 years. We found respiratory symptoms only in one case. The results of laboratory and imaging are shown in Table 2.

**Table 2:** Group 4 features

Patient	Sex	Age	Type of cancer	Symptoms	Leucocytes	Neutrophils	PCR/PCT	LUS	Laboratory
Group 4									
29	F	1	SR	Absent	270	110	22/neg	POS	Neg blood culture
30	M	14	HL	Reduction of VM	290	200	111/0.23	POS	Neg blood culture
31	M	11	ALL	Absent	210	90	93/0.23	POS	Neg blood culture
32	F	3	RMS	Absent	230	0	178/0.32	POS	Neg blood culture
33	F	3	ALL	Absent	1.980	320	20/0.23	POS	Micrococcus luteus in blood culture
34	F	9	ALL	Absent	1.500	580	83/0.12	NEG	Neg galattomannan Neg blood culture
35	F	12	NHL	Absent	860	80	35/neg	NEG	Neg blood culture
36	M	17	ALL	Labial herpes	490	110	259/0.94	NEG	Neg galattomannan Neg blood culture
37	F	12	NHL	Absent	2.090	900	35/neg	NEG	Neg blood culture
38	M	3	WT	Absent	770	80	30/neg	NEG	Neg blood culture

In 50% of cases LUS was found to be pathological (Fig. 2).

None of the ten patients underwent a CXR and in no case was it necessary to perform a chest CT for the benign course of the infection.

Among group 3 and 4, ten patients presented positive LUS and underwent ultrasound follow-up at three and seven days from first LUS showing resolution of the pathological signs in six cases. In the four cases with persistent pathological images at day seven, a further LUS was performed after 1 month, with resolution of pneumonia in all cases.

Statistical analysis showed that LUS, performed in 38 cases, has a specificity of 27/29 93% (95% CI 84–100%), with one finding of pathological ultrasound in an afebrile patient, in the absence of respiratory symptoms and normal inflammation indices. The images matched the CXR ones and were interpreted as leukemic infiltrates at the onset of the disease.

CXR, performed on 16 patients, showed a specificity of 10/12 83% (95% CI 62–100%) displaying pathological images in two cases of onset of leukemia, in apyretic and asymptomatic patients (non-infected patients), likely related to infiltration by leukemic cells.

Sensitivity of LUS for the diagnosis of pulmonary infections was 9/9 100%. In all cases with specific symptomatology and/or CXR or CT images compatible with pneumonia, LUS was positive. Five febrile neutropenic patients in whom there were no respiratory symptoms or comparative radiological tests, all resulted negative at LUS, were excluded from the analysis because we did not know if these exams were true or false negatives. Probably these febrile events indicated an infection without pulmonary involvement.

The sensitivity of CXR was 2/4 50% (95% CI 1–99%).

Pediatric compliance during ultrasound examination: 7/38 patients (18.4%) obtained a score of 0. These children, all younger than 6 years old, were afraid to undergo the ultrasound examination, but the ultrasound was nevertheless completed with informative results. A score of 1 was assigned to 11/38 patients (28.9%), aged 6–17 years; they faced the diagnostic exam with indifference. Finally, 20/38 children (52.6%) aged 2–16 years showed interest and curiosity during the ultrasound exam, obtaining a score of 2 (Table 3).

**Table 3:** Pediatric cancer patients LUS compliance

Sex/age	State of cancer	State	Grade of tolerability
Male, 14 years	Beginning	Apyretic	1
Male, 16 years	Beginning	Apyretic	2
Male, 10 years	No beginning	Apyretic	2
Female, 16 years	No beginning	Apyretic	2
Female, 9 years	No beginning	Apyretic	2
Male, 7 years	No beginning	Apyretic	2
Male, 4 years	No beginning	Apyretic	0
Male, 7 years	Beginning	Apyretic	2
Male, 5 years	Beginning	Apyretic	0
Male, 10 years	No beginning	Apyretic	2
Female, 8 years	No beginning	Apyretic	2
Male, 2 years	Beginning	Apyretic	0
Male, 5 years	Beginning	Apyretic	1
Male, 11 years	Beginning	Apyretic	1
Male, 3 years	Beginning	Apyretic	0
Female, 4 years	Beginning	Apyretic	0
Male, 3 years	No beginning	Apyretic	2
Female, 7 years	No beginning	Apyretic	2
Male, 17 years	Beginning	Apyretic	1
Female, 2 years	Beginning	Apyretic	2
Female, 16 years	No beginning	Apyretic	2
Female, 9 years	No beginning	Pyretic	2
Male, 16 years	No beginning	Pyretic	1
Male, 16 years	No beginning	Pyretic	1
Male, 16 years	No beginning	Pyretic	1
Male, 8 years	Beginning	Pyretic	1
Male, 9 years	No beginning	Pyretic	1
Female, 3 years	No beginning	Pyretic	0
Female, 1 years	No beginning	Pyretic	1
Male, 14 years	Beginning	Pyretic	1
Male, 11 years	No beginning	Pyretic	2
Female, 3 years	No beginning	Pyretic	2
Female, 3 years	Beginning	Pyretic	0
Female, 9 years	No beginning	Pyretic	2
Female, 12 years	No beginning	Pyretic	2
Male, 17 years	No beginning	Pyretic	2
Female, 12 years	No beginning	Pyretic	2
Male, 3 years	No beginning	Pyretic	2

Most patients with score 2 had received tumor diagnosis in a previous admission, while the ones not cooperative or wary were at the onset of their disease. There was no statistically significant difference between febrile and apyretic patients.

## Discussion

Although for a long time it was thought that LUS was not feasible due to the air content, numerous studies in adults and subsequently in children, showed its efficacy for the diagnosis of pneumonia with sensitivity and specificity superior to CXR [17, 19–24]. Therefore, LUS is now identified as a valid substitute for CXR in the course of respiratory tract infections in children with the aim of reducing exposure to ionizing radiation. Moreover, CXR does not allow localization of infection in cancer patients in over 44% of cases [6]. As demonstrated by Gerristen et al. [6] the sensitivity of CT at low doses of radiation is 73% performed on the first day of febrile neutropenia versus the sensitivity of 36% of CXR. Heussel et al. [25] showed that in more than 50% of febrile neutropenic patients with normal CXR, there were signs of pulmonary inflammation on CT.

Ultrasonography is the ideal tool for its speed, non-invasiveness, easy repeatability and simple interpretation of the examination after appropriate training even by non-radiology specialists. This last feature is of increasing importance in relation to the concept of tailored medicine [14]. The “bedside” ultrasonography, performed in the emergency departments or in critical patients, allows a more detailed evaluation of the individual patient obtaining a more patient-based rather than disease-based care approach [14].

In pediatric clinical practice, LUS is becoming an increasingly useful examination; however, no study about its accuracy has ever been performed on the pediatric cancer population, where factors such as type of neoplasia, chemotherapy and thoracic radiotherapy with its pulmonary toxic effects and neutropenia may reduce the specificity and sensitivity of the tests.

Our data indicated that the underlying cancer, the administered therapies, the number of neutrophils did not influence the result, and the specificity was 93%, comparable to that described in the pediatric population for the diagnosis of pneumonia [26]. The sensitivity, even if calculated in a small series, with a no evaluable CI, was 100%.

Our data, with the limit of a small sample, showed that in non-neutropenic febrile patients, LUS has an important role, comparable with the literature on pneumonia cases in pediatric age [26–30]. In this population, LUS showed better diagnostic accuracy than CXR, which had a specificity of 83%, and a sensitivity of 50%, considerably lower than literature data on pediatric population but similar to adult cancer population [6]. LUS showed to have early positivization, even before the onset of clinical respiratory symptoms. This test could therefore be used as a screening tool for pulmonary infections in febrile cancer patients, in order to undertake early specific and targeted treatments or to direct the diagnostic-therapeutic work-up. Moreover, a LUS follow-up allowed to modify or to stop the anti-infection treatment according to the evolution of the process.

In febrile neutropenic patients, despite the absence of respiratory symptoms, it was possible to find lung lesions on ultrasound in 50% of cases, indicating that this method can be an aid technique for the diagnosis of infections during neutropenia, while CXR, with its low sensitivity is not indicated in the suspicion of lung infection during neutropenia. Five out of ten patients presented a positive LUS for pneumonia. These data show that in 50% of patients with febrile neutropenia there is an ongoing lung infection; the latter often resolves thanks to empirical broad-spectrum antibiotic therapy but in a smaller percentage of cases it can evolve towards acute respiratory failure or sepsis [31]. Likely the reason why LUS has greater sensitivity than CXR in this population can be explained by the results obtained by Shah et al. [32]. This study demonstrated that while for pulmonary thickenings of size greater than 1 cm the CXR and the LUS have a similar diagnostic accuracy, in pulmonary thickenings smaller than 1 cm LUS has a clearly superior sensitivity. Therefore, it can be hypothesized that in the patient with a low number of neutrophils and consequent deficient inflammatory response, the infection predisposes to develop subcentimetric thickening, not detectable on CXR. This hypothesis is confirmed by the few data we collected: in 4 out of 5 febrile neutropenic patients (80%), pulmonary thickening on LUS were subcentimetric. This finding could open the door to a new simple diagnostic tool to be used in all neutropenic febrile cancer patients.

As reported by Yan et al. [33] LUS, compared with the chest CT scan, displayed 0.906 sensitivity and 0.661 accuracy, while the chest radiograph displayed 0.793 sensitivity and 0.559 accuracy.

Most patients were compliant to LUS, demonstrating the low invasiveness of the method even in this population. The less cooperative patients were younger than 6 year or patients at the onset of cancer, often frightened by many diagnostic procedures (bone marrow aspirate, lumbar puncture, placement of central venous catheters or excisional biopsy), the hospital environment and the disease itself. Moreover, LUS was efficaciously performed, without the need of sedation, what is not always feasible for CXR or CT.

The possibility of using a tool, such as LUS, which is minimally invasive, repeatable, bedside suitable, radiation free and, at the same time, accurate, as a screening for the early detection of lung infection and monitoring its evolution, alredy applicable and recently proven in the adult, pediatric and neonatal population with suspected respiratory tract infections [34–37] can have a great impact also in the management and outcome of the pediatric cancer patient.

**Fig. 4:**
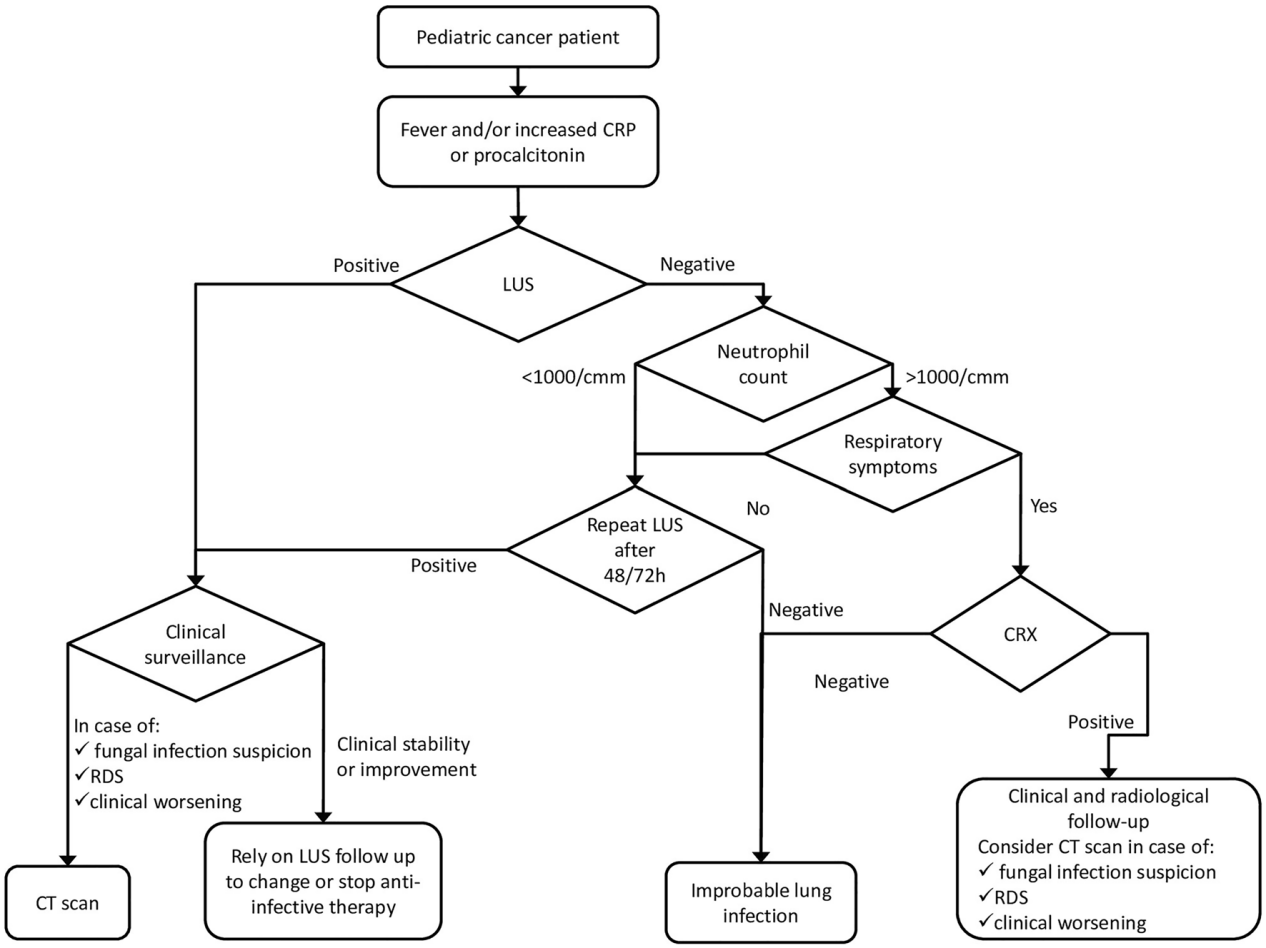
Possible flow chart proposing diagnostic work out

We hypothesize a role for LUS, as a first imaging technique, and propose a possible diagnostic work-up flow chart that includes LUS for the management of patients with signs of infection, with the aim of reducing radiation exposure and inserting a non-invasive and bedside examination (Fig. 4). Obviously, the proposed flow chart does not exempt from individual evaluation and specific clinical management.

## Conclusion

In conclusion, LUS seems to be an accurate and well tolerated method in the diagnosis of pneumonia in febrile neutropenic and non-neutropenic pediatric cancer patients. We therefore believe in the potential of this method for the diagnosis and follow-up of lung infections in pediatric cancer patients. Further studies and larger case series are needed to confirm the hypothesis put forward with this pilot study.

## Supplementary Information

Below is the link to the electronic supplementary material.

Supplementary file1 (MP4 2043 KB)

## Data Availability

General statement: the raw data supporting the conclusions of this article will be made available by the authors, without undue reservation.

## Declarations

### Inclusion of identifiable human data

Generated statement: no potentially identifiable human images or data are presented in the study.
